# Parathyroid, Thyroid and Recurrent Laryngeal Nerve Anatomy in an Indian Rhinoceros (*Rhinoceros unicornis*)

**DOI:** 10.1007/s00268-017-4325-8

**Published:** 2017-11-09

**Authors:** R. Udelsman, S. B. Citino, M. Prasad, P. I. Donovan, D. V. Fredholm

**Affiliations:** 1 Endocrine Neoplasia Institute, Miami Cancer Institute, Baptist Health South Florida, 8900 North Kendall Drive, Miami, FL 33176 USA; 2 Veterinary Medicine, White Oak Conservation, Yulee, FL USA; 3 Department of Pathology, Yale University School of Medicine, New Haven, CT USA; 4 Department of Surgery, Yale University School of Medicine, New Haven, CT USA; 5 Animal Health, Disney’s Animals, Science, and Environment, Bay Lake, FL USA

## Abstract

**Introduction:**

The parathyroid gland was first identified in the Indian rhinoceros in 1849 by Sir Richard Owen. We performed a necropsy in an Indian rhinoceros, recapitulating Owen’s dissection and display what appear to be the initial identification of the recurrent laryngeal nerve in situ and the anatomy and histology of the largest rhinoceros parathyroid glands yet identified.

**Materials and methods:**

Patrick T. Rhino, a 41-year-old Indian rhinoceros was born in 1974. His early years were unremarkable. In 2006, he was donated to White Oak Conservation in Yulee, Florida, where he bred and sustained minor injuries. In his geriatric years, he developed a cataract and degenerative joint disease (DJD). At age 41, he developed progressive ataxia and lameness and was euthanized to minimize suffering when he was unable to stand. ROS, FH, SH and medication history were unremarkable. Physical exam was age and species appropriate. Pre-mortem serum demonstrated: creat 1.8 mg/dL (0.8–2.1), calcium 10.6 mg/dL (9.7–13.1), phos 3.8 mg/dL (2.5–6.7), alk phos 69 U/L (26–158) and intact PTH 44.1 pg/mL (rhinoceros reference range: unknown). Necropsy revealed intervertebral DJD with thoracic spondylosis, which combined with osteoporosis, resulted in thoracic myelopathy and ataxia. The neck block was sent in formalin to the Yale University School of Medicine.

**Results:**

Detailed dissection was performed under loupe magnification. Presumed structures were photographed in situ and biopsied. The thyroid was identified deep to the strap muscles, received its blood supply from the inferior and superior thyroid arteries and was blue in color. The right recurrent laryngeal nerve, identified and photographed in situ for the first time in the rhinoceros, was deep to the inferior thyroid artery and was traced throughout its cervical course. Single parathyroid glands identified on the lateral thyroid lobes received their blood supply from the inferior thyroid arteries and were confirmed histologically. They appear to be the largest parathyroids yet identified in the rhinoceros with estimated weights of 6,280 and 11,000 mg, respectively. Although the etiology of the parathyroid gland enlargement is unknown, the specimen has been preserved recapitulating the dissection performed by Sir Richard Owen.

**Conclusion:**

The parathyroids, thyroid and recurrent laryngeal nerve were identified in an Indian rhinoceros. This appears to be the first display of the rhinoceros recurrent laryngeal nerve in situ, and the parathyroid glands are the largest yet identified in the rhinoceros.

## Introduction

**Fig. 1:**
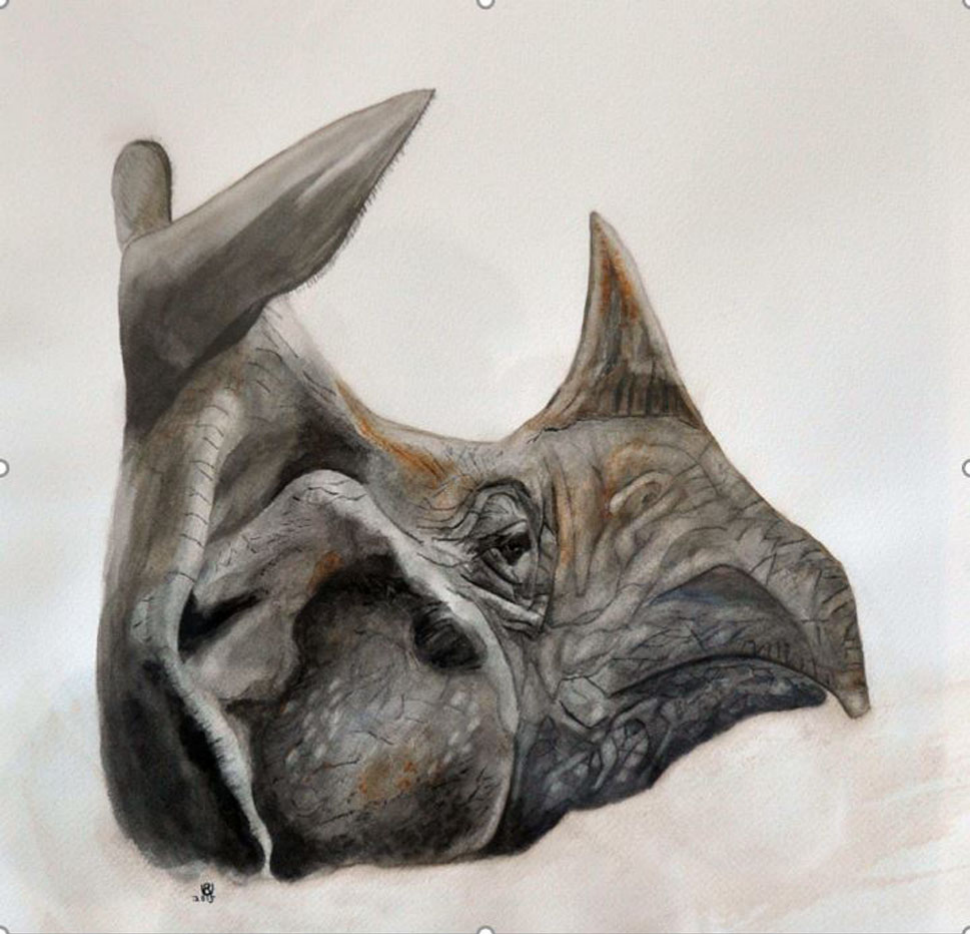
Indian rhinoceros, lateral view of the head demonstrating the classic single horn and overriding upper lip. Water color on paper by Robert Udelsman, 2015

There are five living species of rhinoceros (Rhinocerotidae), whereas six other species are extinct. The remaining species include the White, Black, Javan, Sumatran and Indian rhinoceroses (rhinoceros or rhinoceri). The Indian rhinoceros (*Rhinoceros unicornis*) can still be found in Nepal and North-Eastern India [1]. The Indian rhinoceros like the Javan rhinoceros have a single horn unlike the other three sub-species all of which have two horns, and the Indian rhinoceros has an overriding upper lip as demonstrated in Fig. 1.

**Fig. 2:**
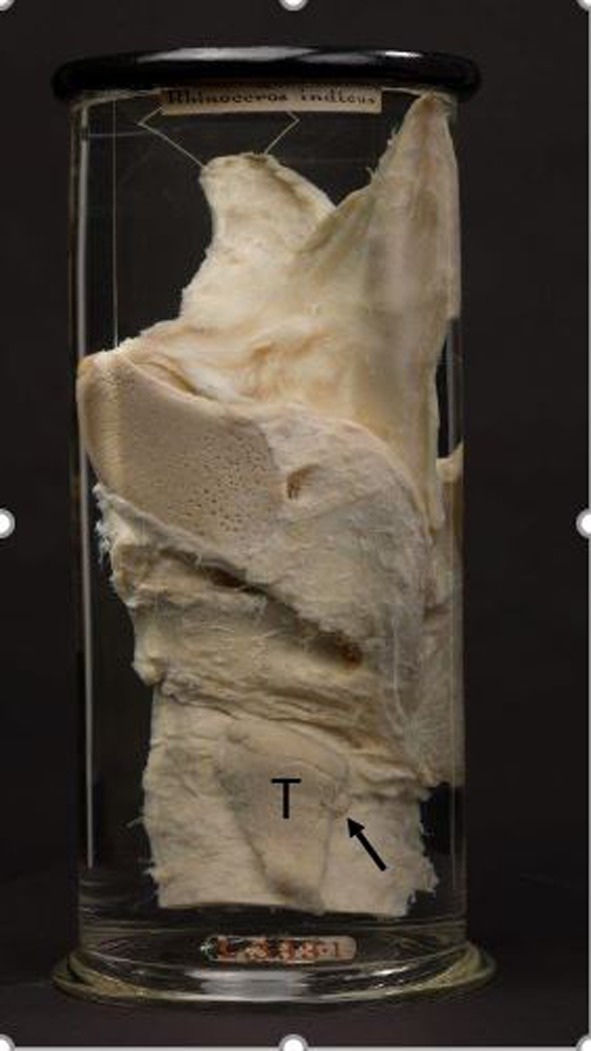
Photograph of original Indian rhinoceros specimen prepared by Sir Richard Owen in 1849 and stored in formalin in the Hunterian Museum, Royal College of Surgeons, London. Photograph courtesy of Bruce Simpson, Curator

Sir Richard Owen performed a dissection upon an Indian rhinoceros in 1849, delivered his findings in 1850 to the Zoological Society of London and published his description stating “a small compact glandular body was attached to the thyroid at the point where the veins emerge.” in 1862 [2]. Although he never performed histology, the original specimen was preserved and can be viewed in the Huntarian Museum of the Royal College of Surgeons in London as shown in Fig. 2. The original description of the parathyroid gland was attributed for many years, to the Swedish medical student Ivor V. Sandström, at the University of Uppsala where he independently rediscovered the parathyroid gland in a variety of species including the first description in humans and drew exquisite illustrations describing the parathyroid gland anatomy, histology and blood supply. His original and detailed manuscript was rejected by the editorial staff of a well-recognized German journal, and it was subsequently published in a regional Swedish journal [3]. Owen’s and Sandstorm’s manuscripts are historical classics.

Over the ensuing years occasional publications emerged describing parathyroid glands in the Indian rhinoceros and other Rhinocerotidae sub-species [4, 5]. The rhinoceros usually has a single parathyroid gland located on the lateral and posterior surface of the respective thyroid lobe. The blood supply has been described as originating from both the inferior and superior thyroid arteries [4]. To the best of our knowledge, the in situ anatomy of the recurrent laryngeal nerve has not been demonstrated in the rhinoceros.

## Materials and methods

Due to the historical significance of the Indian rhinoceros, I contacted several exotic and endangered animal sanctuaries and received a receptive response from Scott Citino, DVM, at White Oak Conservation in Yulee, Florida.

Patrick T. Rhino, *Rhinoceros unicornis* (one-horned), an Indian rhinoceros was captive born on January 30, 1974. His 28-year-old mother and 19-year-old father were maintained in captivity at the Assam State Zoo Botanical Garden in Assam, India. The parents had been on loan to the Smithsonian National Zoological Park (National Zoo) in Washington, D.C. Patrick’s early years were unremarkable. He was on loan to the Bronx Zoo (October 30, 1975–June 15, 1989) and the Toronto Zoo (June 16, 1989–April 11, 2006). He was donated to the Yulee, White Oak Sanctuary on April 11, 2006, where he remained for the rest of his life. His care was meticulously maintained and recorded in his medical record.

During his stay at Yulee he was bred to “Mechi” (2006–2009) and “Chitwan” (2009–2011). During breeding, he sustained minor injuries including a cracked horn, superficial abscess, corneal abrasion and minor bites and bruises. In his geriatric years, he was noted to have a left cataract and right eye opacity. He had a chronic history of degenerative joint disease (DJD) including vertebral spondylosis and intervertebral DJD. He developed progressive ataxia and lameness in his right front and rear limbs which eventually became refractory to medical therapy until the point that he was unable to stand and support himself. Due to his diminished quality of life, he was euthanized on October 12, 2015. A formal necropsy was performed at the Yulee sanctuary by Scott B. Citino, DVM, and the neck block was sent to Robert Udelsman, MD, MBA at the Yale University School of Medicine.

## Results

Immediately prior to death, a full set of blood hematology and chemistry data were obtained. They were essentially within the normal range for the rhinoceros. Selected laboratory values are shown in Table 1. The serum creatinine, calcium, phosphorus and alkaline phosphatase were all normal.

**Table 1:** Serum laboratory values in an Indian rhinoceros

Reference ranges		
Creatinine	1.8 mg/dL	(0.8–2.1)
BUN	27 mg/dL	(8.0–24)
Calcium	10.0 mg/dL	(9.7–13.1)
Phosphorus	3.3 mg/dL	(2.5–6.7)
Alk. Phosphatase	69 U/L	(26–158)
WBC	8.44 cells/μL	(3.9–9.8)
HCT	33.2%	(28.2–49)

Necropsy findings: Remarkable for the paucity of adipose tissue and generalized muscle wasting most notably of the pelvic limbs, neck and back. The integument demonstrated numerous abrasions. The horn was severely worn. The musculoskeletal system demonstrated moderate to marked osseous proliferation/spondylosis in the majority of the cranial thoracic vertebrae; several disk spaces were narrowed and calcified. In the gastrointestinal track, numerous cestode segments were found throughout the small bowel. The eyes demonstrated bilateral mild-to-moderate axial corneal opacification and incipient cataract formation.

Necropsy interpretation was significant for thoracic spondylosis with apparent nerve entrapment, suspected intervertebral DJD and severe osteoporosis causing thoracic myelopathy and resultant ataxia with the inability for the rhinoceros to stand.

**Fig. 3:**
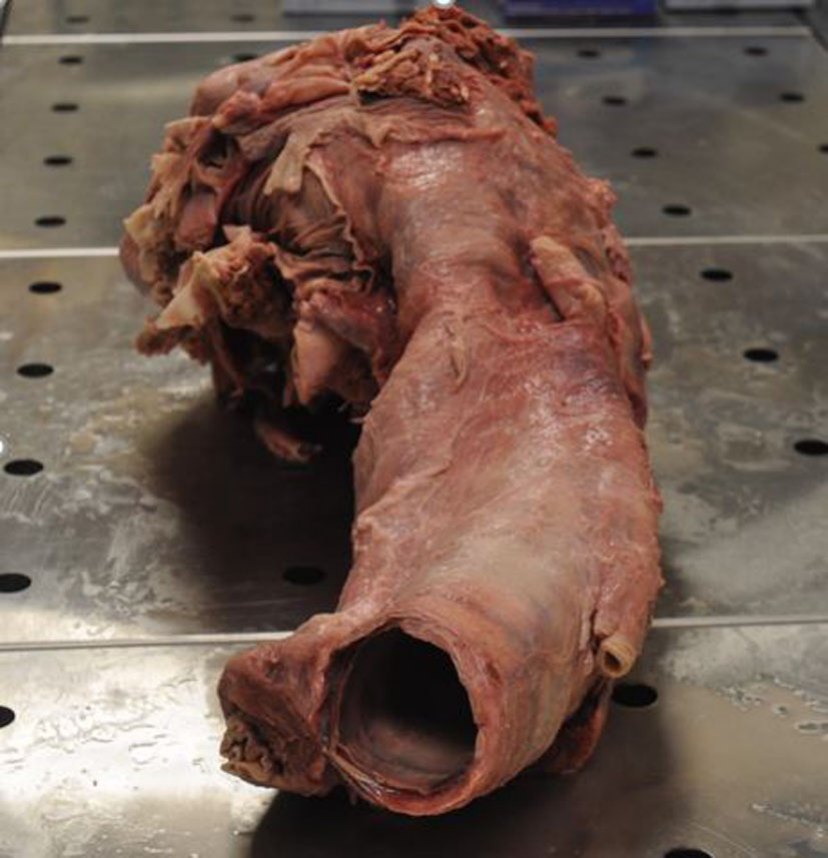
Neck block received from the necropsy of an Indian rhinoceros. The trachea is seen with a large lumen and posterior to it is the esophagus

**Fig. 4:**
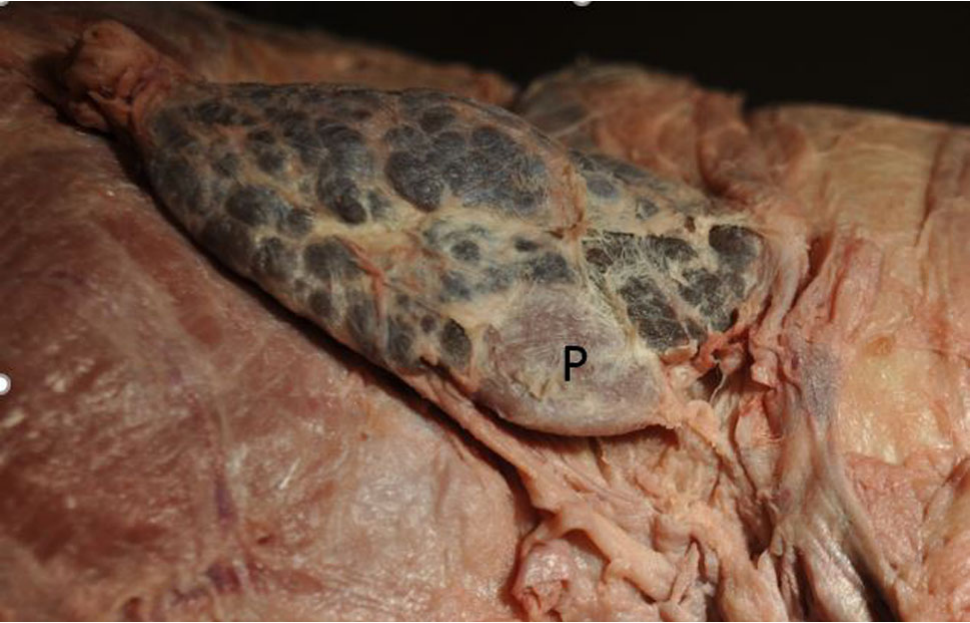
Right thyroid lobe of an Indian rhinoceros. Note the blue color and the right parathyroid gland (P) located at the inferior border of the thyroid lobe. *P* parathyroid gland

Specimen received: We received the neck block consisting of the trachea and neck region (Fig. 3). The skin had been removed. It had been transported in formalin and was slightly compressed in the container resulting in cervical angulation. Multiple photographs were taken during each phase of dissection. The specimen was dissected over a period of 1 month. The trachea had a diameter of 8 cm in its inferior aspect, which would have been well above the carina. Posteriorly the esophagus was compressed and measured 2 cm in diameter. Bilateral common carotid arteries were present. The length of the carotid arteries received was 23 cm. In addition, the internal jugular veins and vagi were present. The vagi were 1 cm in diameter. The strap muscles were present anteriorly and were dissected off the thyroid. The thyroid was blue in color, similar to the thyroid one sees in human patients who had received tetracycline in childhood. Present were a right thyroid lobe, isthmus, left lobe and diminutive pyramidal lobe. The right lobe was 5 × 6.5 × 1 cm in size (Fig. 4). The left lobe was similar in size and appearance.

**Fig. 5:**
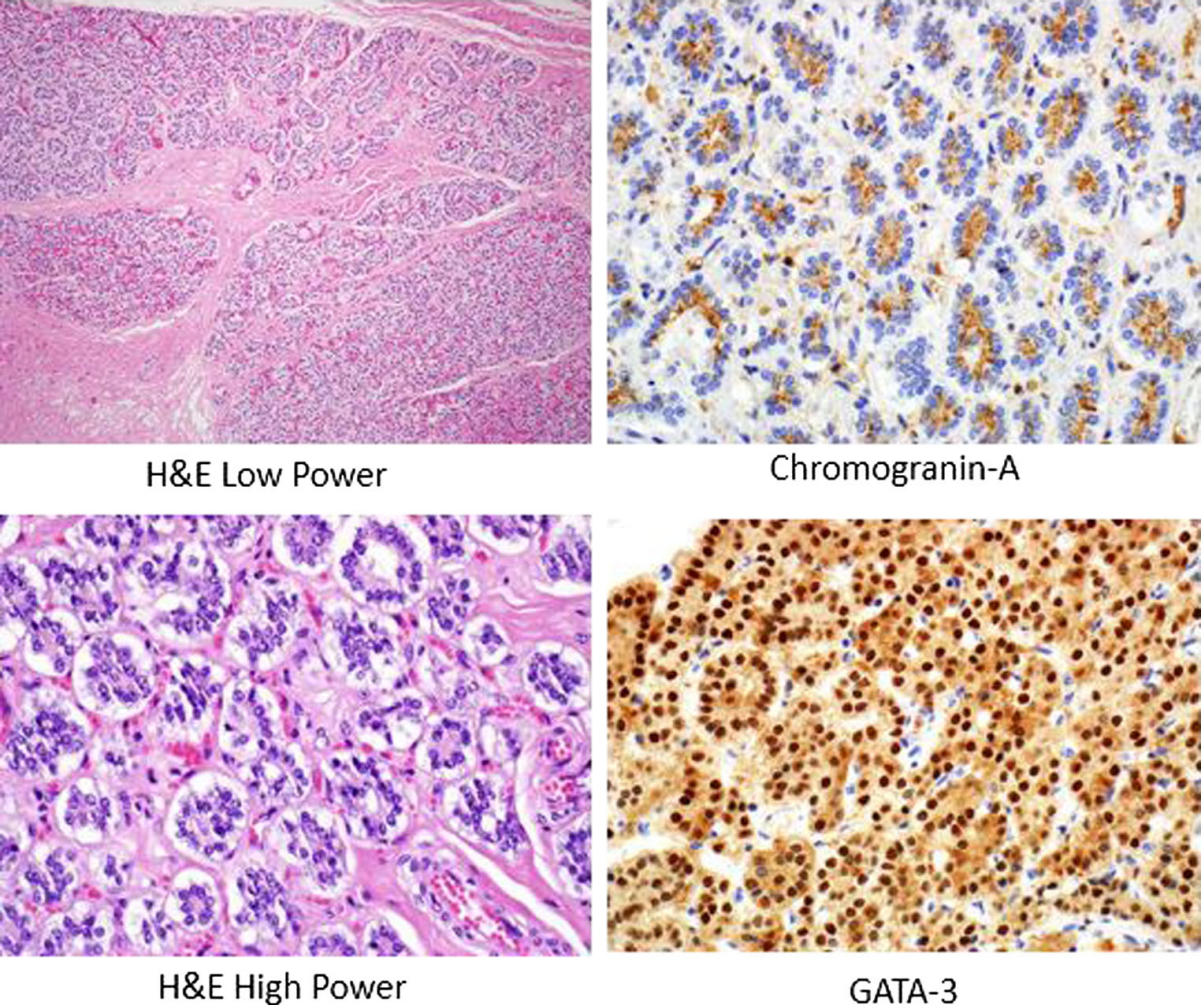
Histologic sections obtained from the left parathyroid gland in an Indian rhinoceros. The tissue was stained with hematoxylin and eosin, and immunohistochemistry was performed for chromogranin A and GATA-3 showing cytoplasmic and nuclear expression, respectively

After the strap muscles were split in the midline and dissected bilaterally, a detailed dissection was performed to identify potential parathyroid tissue from the level of the hyoid bone (superiorly) to the mediastinum (inferiorly) and bilaterally beyond the carotid sheaths. All suspicious nodules were dissected, photographed and biopsied. Following six separate dissection episodes with sequential histologic confirmation, one parathyroid gland was identified associated with both the right and left thyroid lobes. The locations of the glands were meticulously maintained, photographed and preserved intact. The right parathyroid gland measured 2.0 × 1.5 × 0.5 cm and the left 2.5 × 1.5 × 0.7 cm. In order to maintain the specimen intact and in continuity, direct weights of the parathyroid glands could not be obtained. However, by employing the volume calculation of an ellipsoid, where an ellipsoid volume *V* = 4/3 π *abc* (*V* = volume and *abc* = the three axes) it was possible to calculate the parathyroid volume and extrapolate their weights because 1 cm^3^ of water weighs 1 gm and a biologic structure like a parathyroid gland has a density similar to that of water [6]. Accordingly, the right parathyroid gland weighed 6.28 gm (6280 mg) and the left 11.00 gm (11,000 mg). Histologic confirmation of the parathyroid glands is demonstrated in Fig. 5. The parathyroid glands were comprised of lobules containing bland endocrine cells arranged in a tuboglandular formation with small lumina. Unlike human parathyroid glands, intraglandular adipose tissue was not identified. The parathyroid cells expressed chromogranin A in the cytoplasm and showed nuclear expression of the transcription factor GATA-3 confirming parathyroid origin.

**Fig. 6:**
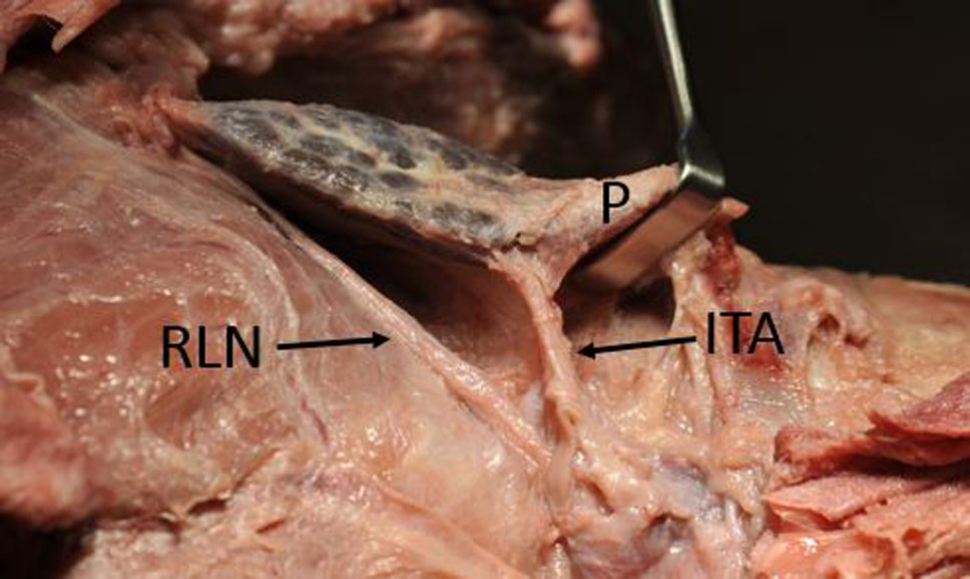
Right thyroid lobe in an Indian rhinoceros. The lobe is elevated with a retractor demonstrating the right recurrent laryngeal nerve (RLN), inferior thyroid artery (ITA) and the parathyroid gland (P)

In addition, on the right side, the recurrent laryngeal nerve was identified and photographed in situ (Fig. 6). It entered the tracheoesophogeal groove and continued superiorly deep to the inferior thyroid artery. The thyroid blood supply was from the superior and inferior thyroid arteries. The parathyroid blood supply was exclusively from the inferior thyroid artery. The inferior thyroid arteries were traced deep to the common carotid arteries and descended inferiorly presumably to the thyrocervical trunk and subclavian arteries which were not included in the specimen received. The superior thyroid artery branches were also present bilaterally; however, their continuity to their origin from the external carotid artery had been disrupted prior to receipt of the specimen.

Additional structures identified and confirmed histologically were the thyroid gland, epiglottis, undescended cervical thymus, lymph nodes, vagus nerves, common carotid arteries and internal jugular veins. The common carotid artery showed no evidence of atherosclerotic disease in this geriatric rhinoceros presumably due to a herbivore diet.

Stored frozen serum was obtained and shipped frozen for parathyroid hormone (PTH) analysis employing the Roche intact PTH platform (human reference range 10–69 pg/ml). The serum PTH in Patrick T. Rhino was 44.1 pg/ml (rhinoceros reference range: unknown).

## Discussion

**Fig. 7:**
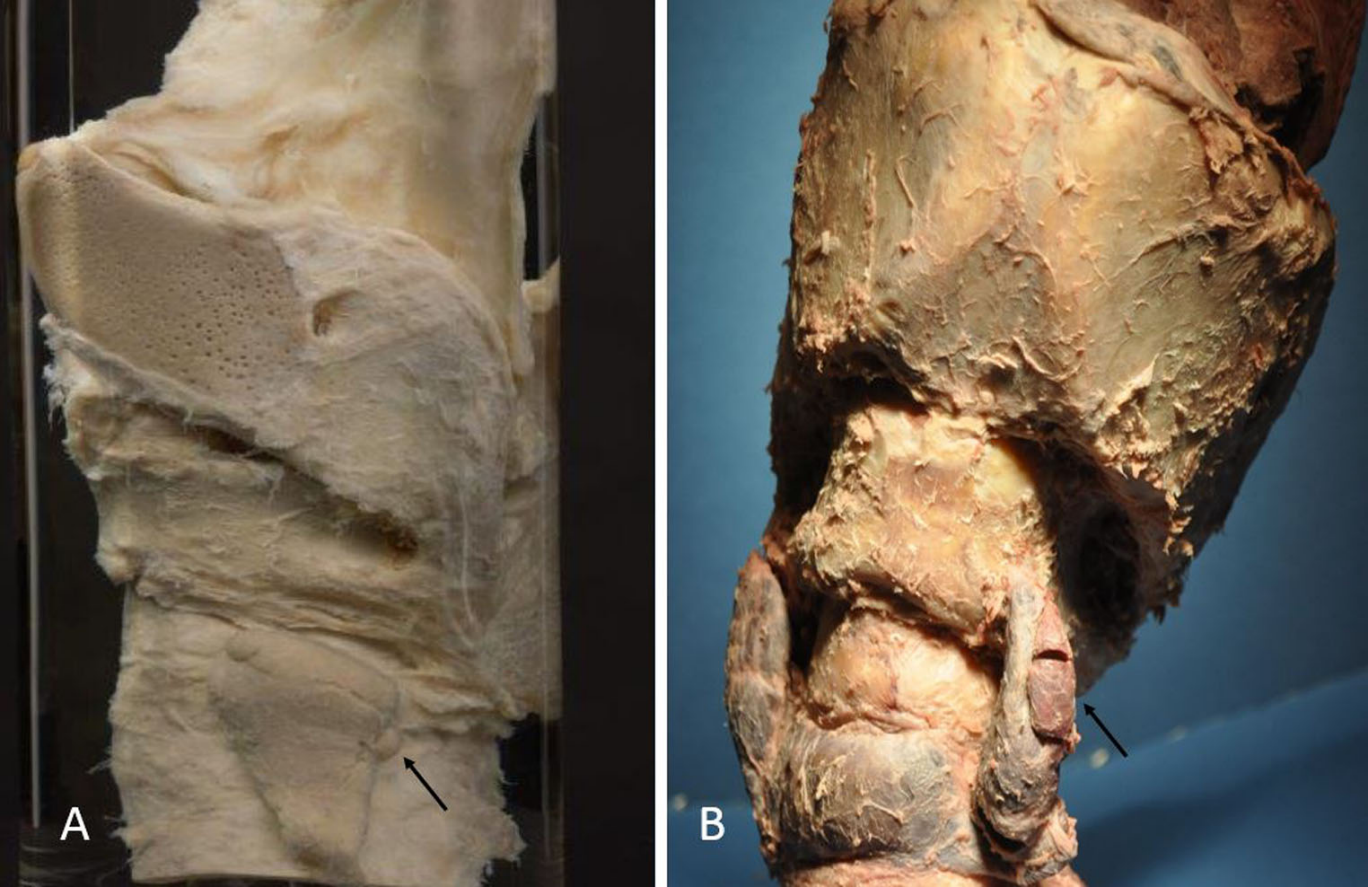
Side-by-side comparison of the original Indian rhinoceros preparation by Sir Richard Owen and the current specimen. **a**  Owen’s specimen demonstrating the neck block and left thyroid lobe. The black arrow points to the left parathyroid gland. **b** Current specimen of the neck block, left thyroid lobe, isthmus and right thyroid lobe. The black arrow points to the left parathyroid gland. The tissue obtained from the biopsy site in the upper third of the parathyroid gland is shown microscopically in Fig. 5. Note the marked difference in the size of the left parathyroid glands as indicated by the black arrows. Photograph of the Owen’s specimen courtesy of Bruce Simpson, Curator, Hunterian Museum, Royal College of Surgeons, London

In 1976, AJE Cave of the Zoological Society of London recorded the sizes, locations and drew illustrations of 17 parathyroid glands in Rhinocerotidae sub-species. Most Rhinocerotidae have two parathyroid glands, one on each of the lateral thyroid lobes. However, extra-numerary and intrathyroidal parathyroid glands have been identified in the rhinoceros [4]. Parathyroid gland sizes in the rhinoceros have ranged from 4 to 13 mm in maximal length [4], whereas the maximal length in Patrick T. Rhinoceros was 25 mm. This difference in size is substantial and results in a change in volume by an order of magnitude. The marked enlargement of one of the parathyroid glands found in the current dissection is demonstrated in Fig. 7 in which it is shown in comparison with the original dissection performed by Sir Richard Owen. This size discrepancy is further demonstrated in Table 2 based on size measurements in 12 rhinoceros reported by Cave [4]. Seventeen parathyroid glands were identified in 7 Indian, 2 Sumatran, 2 White and 1 Black rhinoceros. Although Cave only reported 1–2 axes, detailed published illustrations allow estimation of the missing data and the parathyroid weight can be calculated based on the ellipsoid volume calculation. Accordingly, the mean estimated parathyroid weight in the rhinoceros reported by Cave is 235.24 mg (range: 16.76–387.04).

**Table 2:** Estimated parathyroid gland weights in the rhinoceros

Axes (A, B, C) (mm)						
Para	Case	Species	*A*	*B*	*C*	Weight (mg)
1	1	Indian	11	11	0.5 (e)	253.42
2	2	Indian	10	12	0.5 (e)	251.33
3	2	Indian	12	11 (e)	0.7 (e)	387.04
4	3	Indian	12	11 (e)	0.7 (e)	387.04
5	3	Indian	12	12 (e)	0.5 (e)	301.59
6	4	Indian	12	12 (e)	0.5 (e)	301.59
7	5	Indian	12	10	0.5 (e)	251.33
8	6	Indian	11	11 (e)	0.3 (e)	152.05
9	6	Indian	5	4	0.2 (e)	16.76
10	7	Indian	13	11	0.5 (e)	299.50
11	7	Indian	12	12	0.5 (e)	301.59
12	8	Sumatran	12	4	0.3 (e)	60.32
13	9	Sumatran	9	8	0.3 (e)	90.48
14	9	Sumatran	9	8	0.3 (e)	90.48
15	10	White	12	12	0.5 (e)	301.59
16	11	White	11	12	0.5 (e)	276.46
17	11	White	11	12	0.5 (e)	276.46
–	12	Black	Size not reported			

*e* estimate based on illustrations by Cave [4]

In previous publications, the authors indicated that the inferior thyroid artery of the rhinoceros was a branch of the common carotid artery [4]. This was not the case in this dissection where the inferior thyroid blood supply to each parathyroid gland was from an arterial inflow independent of the common carotid artery, presumably the thyrocervical trunk.

The current report is based on a single Indian rhinoceros and has limitations. The estimates of parathyroid gland weights are based on a mathematical formula and not direct measurements because we maintained the dissection intact. Nonetheless, based on the photographs presented these estimates appear to be reasonable. It would also have been ideal if we could have measured vitamin D levels and compared them to normative data in the rhinoceros to rule out the remote possibility of secondary hyperparathyroidism. However, it is highly unlikely that Patrick had secondary hyperparathyroidism because he spent the last 9 years of his life in an expansive outdoor pen during the day in Yulee, Florida.

The parathyroid glands detected in Patrick, the Indian rhinoceros, were substantially larger than the parathyroid gland identified by Sir Richard Owen or any other parathyroid identified in any rhinoceros sub-species to date. There is no clear explanation why these parathyroid glands were an order of magnitude larger than any rhinoceros parathyroid gland yet described. Patrick T. Rhino did not demonstrate any apparent signs of primary or secondary hyperparathyroidism, and his pre-mortem serum creatinine, PTH, calcium and alkaline phosphatase levels were normal. Furthermore, his degree of DJD is commonly seen in the geriatric rhinoceros and his life span was typical for a captive rhinoceros. Although we cannot offer a clear explanation for these remarkably enlarged rhinoceros parathyroid glands, I have maintained the specimen in formalin for other investigators.
